# L-serine deficiency: on the properties of the Asn133Ser variant of human phosphoserine phosphatase

**DOI:** 10.1038/s41598-024-63164-y

**Published:** 2024-05-30

**Authors:** Loredano Pollegioni, Barbara Campanini, Jean-Marc Good, Zoraide Motta, Giulia Murtas, Valeria Buoli Comani, Despina-Christina Pavlidou, Noëlle Mercier, Laureane Mittaz-Crettol, Silvia Sacchi, Francesco Marchesani

**Affiliations:** 1 Department of Biotechnology and Life Sciences, University of Insubria, via J.H. Dunant 3, 21100 Varese, Italy; 2 Department of Food and Drug, University of Parma, 43124 Parma, Italy; 3 Division of Genetic Medicine, Lausanne University Hospital (CHUV) and University of Lausanne, Lausanne, Switzerland; 4 Department of Epileptology, Institution of Lavigny, Lavigny, Switzerland; 5 Department of Medicine and Surgery, University of Parma, 43121 Parma, Italy

**Keywords:** Serine deficiency, Phosphorylated pathway, Genetic disease, Structure–function relationships, Biochemistry, Neuroscience

## Abstract

The non-essential amino acid L-serine is involved in a number of metabolic pathways and in the brain its level is largely due to the biosynthesis from the glycolytic intermediate D-3-phosphoglycerate by the phosphorylated pathway (PP). This cytosolic pathway is made by three enzymes proposed to generate a reversible metabolon named the “serinosome”. Phosphoserine phosphatase (PSP) catalyses the last and irreversible step, representing the driving force pushing L-serine synthesis. Genetic defects of the PP enzymes result in strong neurological phenotypes. Recently, we identified the homozygous missense variant [NM_004577.4: c.398A > G p.(Asn133Ser)] in the *PSPH*, the PSP encoding gene, in two siblings with a neurodevelopmental syndrome and a myelopathy. The recombinant Asn133Ser enzyme does not show significant alterations in protein conformation and dimeric oligomerization state, as well as in enzymatic activity and functionality of the reconstructed PP. However, the Asn133Ser variant is less stable than wild-type PSP, a feature also apparent at cellular level. Studies on patients’ fibroblasts also highlight a strong decrease in the level of the enzymes of the PP, a partial nuclear and perinuclear localization of variant PSP and a stronger perinuclear aggregates formation. We propose that these alterations contribute to the formation of a dysfunctional serinosome and thus to the observed reduction of L-serine, glycine and D-serine levels (the latter playing a crucial role in modulating NMDA receptors). The characterization of patients harbouring the Asn133Ser PSP substitution allows to go deep into the molecular mechanisms related to L-serine deficit and to suggest treatments to cope with the observed amino acids alterations.

## Introduction

L-serine (L-Ser) is a non-essential amino acid, since it is available from different sources, i.e. absorption from dietary proteins and degradation from proteins and phospholipids. Notably, it is also synthesised by two main pathways: from glycine via serine hydroxymethyltransferase (SHMT, both in mitochondria and cytosol) and from glucose via the cytosolic phosphorylated pathway (PP). This latter pathway converts the glycolytic intermediate D-3-phosphoglycerate (3PG) into L-Ser through three sequential reactions catalysed by the enzymes 3-phosphoglycerate dehydrogenase (PHGDH, EC 1.1.1.95), phosphoserine aminotransferase (PSAT, EC 2.6.1.52) and phosphoserine phosphatase (PSP, EC 3.1.3.3)^1^. The expression of these three enzymes is coordinated in many tissues: we have recently reported that they co-localize in cytoplasmic clusters of differentiated astrocytes, yielding a novel metabolon we named the serinosome^2^. L-Ser biosynthesis has been studied extensively for its relevance to cellular proliferation: it provides formyl groups for purine synthesis and methyl groups for pyrimidine synthesis. However, the PP is particularly important in the central nervous system since it can be converted by serine racemase into D-serine (D-Ser)^3^, the main co-agonist of NMDA receptors in the hindbrain^4^. Because of the limited permeability of L-Ser through the blood–brain barrier^5^, endogenous synthesis is required. L-Ser is mainly produced in astrocytes, indicating that serine can be considered an essential amino acid for neurons^6^. The neurological phenotypes associated with genetic defects of either one of the PP enzymes highlight its relevance in the central nervous system, see below.

**Figure 1:**
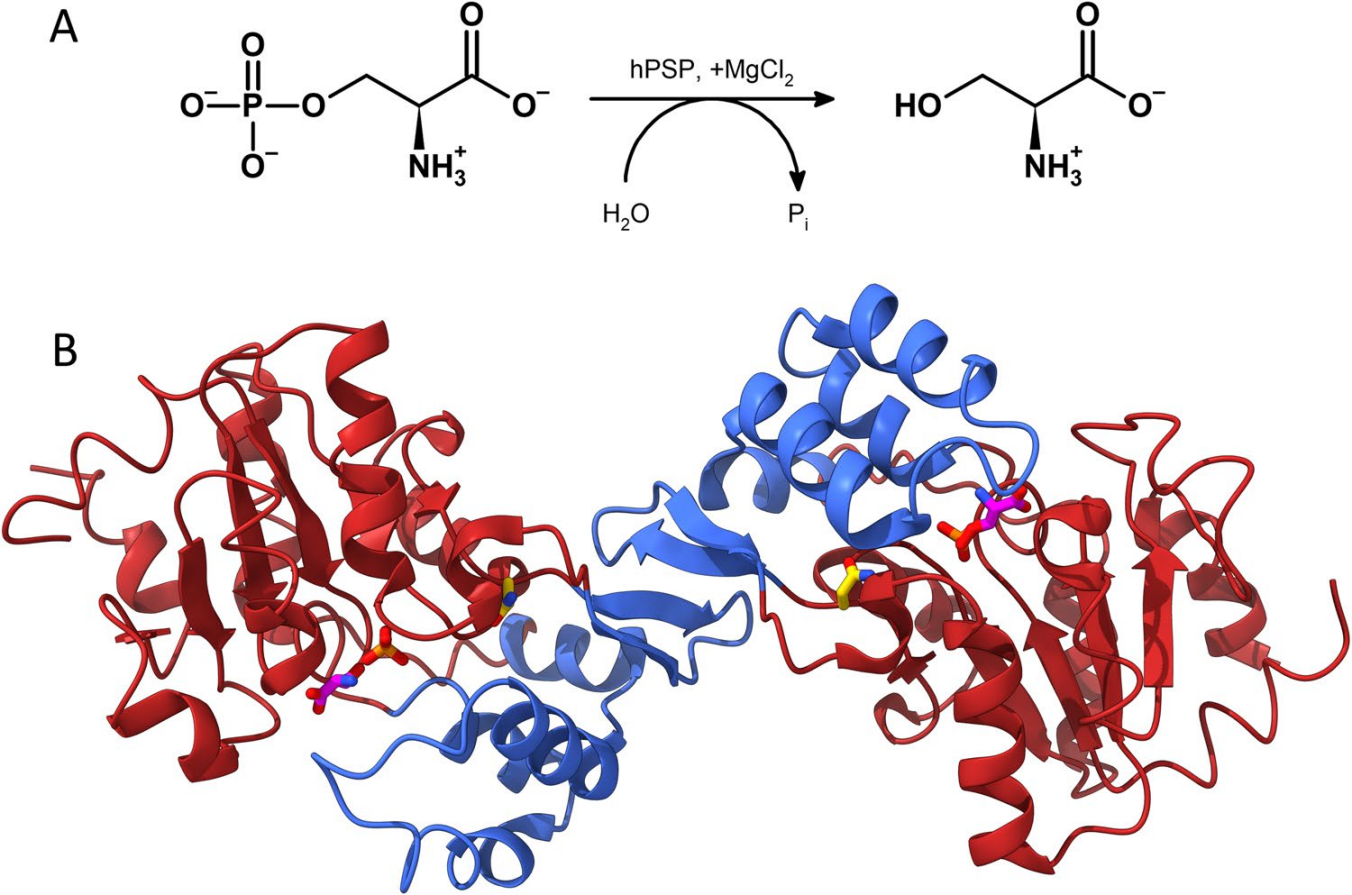
(**A**) Reaction catalysed by PSP: 3-phosphoserine (3-PS) is hydrolyzed into L-Ser and phosphate. (**B**) Three-dimensional structure of PSP (PDB code 1L8O) with L-Ser, phosphate and Asn133 residue shown in capped stick mode. The Rossmann domain is shown in red, while the subdomain is shown in blue.

Among the PP enzymes, kinetic studies recently highlighted the key role of PSP catalysed reaction (Fig. 1A) as the driving force pushing the PP in the direction of L-Ser production^2^. PSP is a member of the haloacid dehalogenase-like superfamily^7^. Native human PSP purified from brain can dephosphorylate both L- and D-phosphoserine, with higher affinity for the L-enantiomer. L-Ser, a known inhibitor of the isolated PSP, does not exert any effect on the flux through the PP unless the enzyme activity is severely impaired by inactivating substitutions^2,8^. The enzyme is Mg^2+^-dependent^9,10^. Other di- or trivalent ions inhibit enzymatic activity (e.g., Ca^2+^, Mn^2+^, Al^3+^) alongside antipsychotic drugs such as trifluoperazine and chlorpromazine, and antiepileptics such as phenytoin and phenobarbital. On the other hand, benzodiazepines, such as diazepam and chlordiazepoxide, play an activating effect on PSP^11^. Human PSP contains 225 amino acids (for a 25 kDa mass) and forms a 50 kDa homodimer under physiological conditions. The resolution of the crystal structure of PSP confirmed its dimeric state (Fig. 1B). Each monomer consists of two major domains: one domain resembles a Rossmann-fold and the second one consists of the dimer-interface part and a four-helix bundle^12^.

PSP defects have been linked to the onset of neurological disorders, and a number of SNPs altering the enzymatic activity have been identified, for a review see^13,14^. A patient showed the replacement of a well conserved aspartate (Asp32Asn) and of an extremely conserved methionine (Met52Thr)^15^: the specific activity values of these variants were ∼ 50% and 3% of the wild-type enzyme, respectively. Interestingly, the patient showed decreased cerebrospinal fluid serine levels, while phosphoserine and glycine levels were normal^15^. In a family in which individuals had moderate to profound intellectual disability and seizures, the Ala35Thr PSP substitution was identified: the specific activity was ∼ tenfold lower than that of the wild-type enzyme. On the other hand, in patients with schizophrenia, PSP activity was significantly higher than in controls, especially in male subjects: PSP activity negatively correlated with plasma D-serine and glycine (Gly) concentrations and D-serine/total serine ratio, and positively, albeit weakly, with plasma L-Ser concentration, especially in male patients^16^. PSP upregulation was also observed in melanoma, neuroblastoma and hepatocellular carcinoma^17–19^. PSP knockdown tumour cells showed an increase in 2-hydroxyglutarate level^18^, which inhibits the TET family of DNA demethylases and the Jumonji family of histone demethylase, which impact on gene expression.

Two PSP variants at position 133 are reported in gnomAD, namely the Asn133Ser and Asn133His. The first one has been classified as benign in amelogenesis imperfecta by ClinVar and mainly present in the European population. This residue is fully conserved in 19 members of the PSP family. Asn133 substitution affects the enzymatic activity: a 30% increase was reported for the Asn133Asp variant and a 70% decrease for the Asn133Ala one. Its role has been related to both active site dynamics (playing a role in structural integrity of the four-helix bundle during the rearrangement between the open and the closed conformations) and dimer stabilisation^20^. Recently we identified by whole-exome sequencing the homozygous missense variant [NM_004577.4: c.398A > G p.(Asn133Ser)] in the human PSP encoding gene in two siblings with a neurodevelopmental syndrome, a myelopathy (spastic paraplegia) and a low L-Ser plasma level compared to healthy controls. The clinical phenotype corresponds well to the one described by^21^ concerning a patient with two substitutions (Val44Gly and Gly141Ser).

In this work, we report on the investigations of the biochemical alterations in human PSP properties of the recombinant Asn133Ser variant, on the L-, D-Ser and glycine levels in the serum and fibroblasts of the two affected siblings, as well as on the expression of the three PP enzymes and their ability to generate the serinosome. The overall results allow a deep insight the relationships between the biochemical properties of PSP and the alterations in serine levels under pathological conditions, this to pave the way to targeted therapeutic approaches to PSP deficiency disorders and to alterations related to the serinosome.

## Methods

### Genetic analysis

With appropriate informed consent of the parents, exome sequencing was conducted on NextSeq 500 sequencing from Illumina using the Comprehensible library from Twist Biosciences^®^, on genomic DNA extracted from the proband’s leukocytes. The raw NGS reads were aligned to the human reference genome GRCh37/hg19 with Novoalign (Novocraft Technologies; v4.02.02) and bioinformatics analysis was carried out using an in-house pipeline (v.8.3). Variant calling followed the GATK Best Practices recommendations (https://gatk.broadinstitute.org/). CNV analysis was based on the ExomeDepth tool^22^. The analysis targeted virtual panels of genes related to epilepsy (40 genes), neurodevelopmental disorders (1832 genes) and hereditary spastic paraplegia (148 genes). Familial segregation analysis was performed by Sanger sequencing on DNA extracted from leukocytes.

### Protein expression and purification

The gene encoding human PSP, cloned in the NdeI/BamHI sites of the pET28a vector, was used to express the variant enzyme in *E. coli* BL21(DE3) cells (Novagen^®^, Merck, Darmstadt, Germany)^23^. The plasmid carrying the mutation coding for the Asn133Ser substitution was prepared by GenScript. The proteins were expressed and purified using a published procedure^22^. The Asn133Ser variant had a final yield comparable to that of the wt PSP and no special issues were encountered during expression and purification.

### Size exclusion chromatography (SEC)

SEC analyses were performed on an ÄKTA Pure 25 M chromatographic system (GE Health Sciences™, Chicago, IL, USA) equipped with a Superdex 75 Increase 5/150 GL column (GE Health Sciences™) with a mobile phase consisting of 50 mM HEPES, pH 7.0, and 100 mM KCl, at a flow rate of 0.3 mL/min. The separation was run at room temperature and the absorbance of the column effluent was monitored at 280 nm. Proteins were loaded at either 1 or 10 μM. The calibration curve consisted of conalbumin, carbonic anhydrase and lysozyme standard proteins.

### Activity assays

All the assays were carried out at 37 °C in 50 mM HEPES, 100 mM KCl, 3 mM MgCl_2_, pH 7.0. The kinetic parameters were determined by a coupled assay using bacterial serine acetyltransferase (SAT) to detect L-Ser production. The IC_50_ for L-Ser was instead determined by a coupled assay using bacterial purine nucleoside phosphorylase (PNP) to detect phosphate release. Both assays have been described in^23^. Briefly, the SAT-coupled assay contains 170 μM acetyl-CoA, 0.5 mM 5,5'-dithio-bis-(2-nitrobenzoic acid), 430 mU SAT, 12.5 nM PSP and varying concentrations of 3-PS ranging from 10 to 600 μM. The increase in absorbance at 412 nm was used to calculate the rate of product formation using the extinction coefficient of 2-nitro-5-thiobenzoic acid at pH 7.0 (14,100 M^−1^ cm^−1^). The kinetic parameters were calculated by fitting the dependence of the initial rate on 3-PS concentration using the Michaelis–Menten equation (Eq. 1):

1 $$v_{0} = \frac{{k_{cat} \cdot E_{t} \cdot \left[ {3 - PS} \right]}}{{K_{m} + \left[ {3 - PS} \right]}}$$

where v_0_ is the initial velocity, k_cat_ is the turnover number, K_m_ is the Michaelis constant and E_t_ is the total enzyme concentration in the assay mixture. The specific activity was measured at 600 μM 3-PS. One unit is defined as the amount of enzyme that catalyses the conversion of 1 μmol of 3-PS to L-Ser in 1 min at 37 °C.

The PNP-coupled assay contains 100 μM 7-methyl-6-thioguanosine (MESG), 300 mU PNP, 12.5 nM PSP, a 3-PS concentration equal to the K_m_ and varying concentrations of L-Ser between 0.1 and 10 mM. The increase in absorbance at 360 nm was used to calculate the rate of product synthesis using a Δε_360nm_ between MESG and 2-amino-6-mercapto-7-methylpurine of 11,200 M^-1^ cm^-1^. The IC_50_ for L-Ser was calculated by fitting the dependence of the relative activity (f) on the concentration of L-Ser to a binding isotherm (Eq. 2):

2 $$f = \frac{{IC_{50} }}{{\left[ {L - Ser} \right] + IC_{50} }}$$

The stability of PSP was measured by using the SAT-coupled assay. Briefly, 0.95 μM PSP was incubated in 20 mM potassium phosphate, 0.125 mM MgCl_2_, pH 7.0, at 37 °C. Aliquots were withdrawn at time intervals and diluted in the SAT-coupled assay mixture containing 600 μM 3-PS and 3 mM MgCl_2_. The activity at each time point was measured at 37 °C and expressed as a fraction of the initial activity.

The assays were carried out using a double beam spectrophotometer (Cary 4000, Agilent, Santa Clara, CA, USA) with a thermostated cuvette holder.

### In vitro pathway reconstruction

The effect of the Asn133Ser substitution in PSP on the kinetics of the whole PP reconstructed in vitro was assessed as previously described^2^. Briefly, the phosphate produced was measured by an end-point assay using malachite green as detection reagent. The assay mixture in the activity buffer (see above) contained 820 nM PHGDH, 1140 nM PSAT and 120 nM PSP. The substrates were used at the following concentrations: 2 mM L-Glu, 0.3 mM NAD^+^ and 0.54 mM 3PG. All assays were performed in the presence of physiological L-Ser concentration (1 mM). Recombinant human PHGDH and PSAT were expressed and purified as described previously^24,25^.

### Circular dichroism and thermal stability

The far-UV circular dichroism spectra of PSP (a probe of protein secondary structure composition) were collected using a Jasco^®^ 1500 spectropolarimeter (Jasco Co, Tokyo, Japan) equipped with a Peltier system for the temperature control, using a protein concentration of 10 μM in 20 mM potassium phosphate pH 7.0, in a 0.1 cm optical pathlength cuvette. Each spectrum is the average of three acquisitions and was corrected for the buffer contribution. The melting curves (recording the ellipticity at 210 nm) were collected from 25 to 80 °C, with a data pitch of 1 °C, a temperature ramp rate of 5 °C/min, a bandwidth of 1 nm and a digital integration time of 8 s. Data were fitted to Eq. (3)^26^:

3 $$\theta = \theta_{0} + \frac{f}{{1 + e^{{\frac{{T {-} T_{m} }}{k}}} }}$$

where θ is the ellipticity at 222 nm, θ_0_ is an offset, f is the amplitude, k is the slope (with larger values corresponding to shallower transitions) and T_m_ is the melting temperature.

### HPLC analysis

Serum samples were prepared following the procedure previously described in^27^. To precipitate serum proteins, samples were supplemented with HPLC grade methanol to a final concentration of 90% v/v, vortexed for 4 min at room temperature and then centrifuged for 15 min at 16,000×*g* at 4 °C. After an incubation overnight at 55 °C, the resulting pellets were resuspended in 0.2 M trichloroacetic acid. 10 μL of serum or cell samples (see below) were neutralised with NaOH and subjected to pre-column derivatization with o-phthalaldehyde and N-acetyl-L-cysteine. The diastereoisomer derivatives were subsequently separated on a Symmetry C8 reversed-phase column (5 μm, 4.6 × 250 mm, Waters) under isocratic conditions, with a flow rate of 1 mL/min, in a 0.1 M sodium acetate buffer at pH 6.2, with 1% tetrahydrofuran^28^. Identification and quantification of Gly, D- and L- amino acids were performed by comparing peak areas with the ones obtained with external standards. D-enantiomer identity was verified using an enzymatic degradation step by the RgDAAO Met213Arg variant on the pre-derivatization mixture (4 h incubation, 10 μg of enzyme)^29^.

### Cell culture studies

The fibroblasts from affected patients and one healthy control were maintained in DMEM (Invitrogen, 31,885) supplemented with 10% fetal bovine serum, 1% penicillin/streptomycin, and 1% amphotericin B (Euroclone) at 37 °C in a 5% CO_2_ incubator. DMEM medium contains 0.4 mM of both L-Ser and Gly. Fibroblast samples were homogenised in 0.2 M trichloroacetic acid, sonicated (three cycles, 10 s each) and then centrifuged at 13,000*g* for 20 min. The supernatants were stored at − 80 °C for HPLC analysis.

The pellets were solubilized in 1% SDS, sonicated (three cycles, 10 s each) and then centrifuged at 13,000*g* for 20 min. The protein concentration in the supernatants was quantified using the Bradford reagent (500–0205, BioRad). These samples were analysed by Western blot for the presence of PHGDH (rabbit anti-PHGDH, HPA024031, Sigma, dilution 1:1000), PSAT (rabbit anti-PSAT, abin2856767, Antibodies online, 1:1000), and PSP (rabbit anti-PSP, PA5-22003, Invitrogen, dilution 1:1000)^2^. For each sample 40 µg of total proteins were separated by SDS-PAGE and then transferred on a PVDF membrane. The membrane was cut into three pieces: top: 150–50 kDa range, containing PHGDH, 56.7 kDa; center: 36–50 kDa range, containing PSAT and glyceraldehyde-3-phosphate dehydrogenase (GAPDH, used as loading control), 40.5 and 37 kDa, respectively; and bottom: 23–36 kDa range, containing PSP (25 kDa). Each piece was blocked overnight at 4 °C with 4% dried milk in Tris-saline buffer pH 8.0 added of 0.1% Tween 20 and subsequently incubated with primary antibodies (in 2% dried milk in Tris-saline buffer pH 8.0 added of 0.05% Tween 20) for 2 h at room temperature. After extensive washings, the membrane was incubated for 1 h at room temperature with anti-rabbit IgG (Alexa-Fluor Plus 800, 1:20,000 dilution in Tris-saline buffer pH 8.0, 0.05% Tween 20). Membranes were analysed by an Odyssey Fc Imaging system (LI-COR Biosciences) and ImageStudio software and the ImageStudio software: the intensity signal of each sample was normalised by the GAPDH signal (detected using a mouse anti-GAPDH, 1:2000, MA5-15,738 Invitrogen and a mouse IgG IRDye 680 1:5000). The content of each protein was determined using known amounts of recombinant proteins and was related to the mg of total proteins loaded into the gel. Each sample was analysed at least three times (in three different SDS-PAGE runs). The results were analysed using Prism (Graphpad Software Inc.).

To evaluate the stability of PHGDH, PSAT and PSP and assess their rate of degradation, fibroblasts from affected patients and the healthy control were seeded into 6-well plates (2.5 × 10^5^ cells per well) and treated up to 25 h with cycloheximide (CHX, 100 μg mL^−1^; Sigma), a potent inhibitor of protein synthesis, and collected at different times for Western blot analysis. Changes in protein cellular levels were determined by densitometric analysis as above. Data were fit to a single exponential decay equation to estimate the half-life.

### Immunofluorescence and confocal analyses

Fibroblasts were seeded on round coverslips (2.5 × 10^4^ cells each) coated with 0.1% gelatine and fixed in 4% paraformaldehyde when they reached 80% confluency. After block permeation in PBS, 0.2% Triton X-100, 4% horse serum (for 20 min at room temperature), coverslips were incubated overnight at 4 °C with the primary antibodies (diluted in PBS, 0.1% Triton X-100, 4% horse serum) and, after extensive washing, with the secondary antibodies (diluted in PBS, 0.1% Triton X-100) for 1 h at room temperature. Double staining antibody mixtures were prepared as follows: (i) rabbit anti-PHGDH (1 : 1000, HPA024031; Sigma) + mouse anti-PSAT (1 : 250, H00029968-A01; Abnova, Taipei, Taiwan); (ii) mouse anti-PSAT (1:250) + rabbit anti-PSP (1 : 250, PA5-22003; Invitrogen, Waltham, MA, USA); (iii) mouse anti-PHGDH (1:500, raised against the purified recombinant protein; Davids Biotechnologie, Regensburg, Germany) + rabbit anti-PSP (1 : 250). Cells were counterstained with Phalloidin CruzFluorTM488 conjugate (1:1000, sc-363791; Santa Cruz Biotechnology, Dallas, Texas, USA) for 60 min at room temperature to visualise the cytoskeleton or with the fluorescent nuclear probe DRAQ5 (1:500, 62251; Thermo Fisher Scientific, Waltham, MA, USA) for 10 min at room temperature. Alexa Fluor 488 and 546 donkey anti-mouse (A21202, A10036), 546 goat anti-rabbit (A11035) and 647 donkey anti-rabbit (A31573) were used as secondary antibodies (1:1000, Invitrogen)^30^.

Coverslips were mounted using the Vectashield antifade mounting media (H-1700; Vector Laboratories). Stained fibroblasts were imaged using an inverted laser scanning confocal microscope (TCS SP5; Leica Microsystems, Wetzlar, Germany) equipped with a 63.091.25 NA plan apo-chromatic oil immersion objective. Confocal image stacks (10 sections each, optimised thickness) were acquired using the LEICA TCS software with a sequential mode and without saturating any pixel. Images were then prepared by superimposing the stacks acquired with the different channels by the open access Fiji (ImageJ) software. Negative controls (without the primary antibodies) were used to rule out the presence of autofluorescence or nonspecific reactivity during imaging. The overlapping and correlation of fluorescence signals corresponding to the stained PP enzymes, as well as to the nucleus and the cytoskeleton (i.e. actin filaments labelled with phalloidin) were analysed by the JACoP plugin of Fiji (ImageJ) online open source software. Zoomed images were processed subtracting the background and colocalization parameters were calculated: Manders' coefficients M1 and M2, well-established measures of the co-occurrence of couple of signals, which calculate the percentage of total signal from one channel which overlaps with signal from the other (i.e., green over red channel and vice versa) and account for the fraction of the total fluorescence that co-occurs; the Pearsons’ coefficient (*r*, computed selecting a Costes' threshold), a measure of the strength of the linear relationship between the two channels. M1 and M2 coefficients were determined by adjusting the threshold of the considered channels based on the detected signals.

### Ethics approval and consent to participate

All methods were carried out in accordance with relevant guidelines and regulations. This work is considered as a “case study” and for this reason the ethics committee waived the need for approval because this did not fall within the scope of Swiss national law related to research on human beings. Informed consent to publish (clinical description and photos) was obtained from the parents of the patients.

## Results

### Clinical evaluation

**Figure 2:**
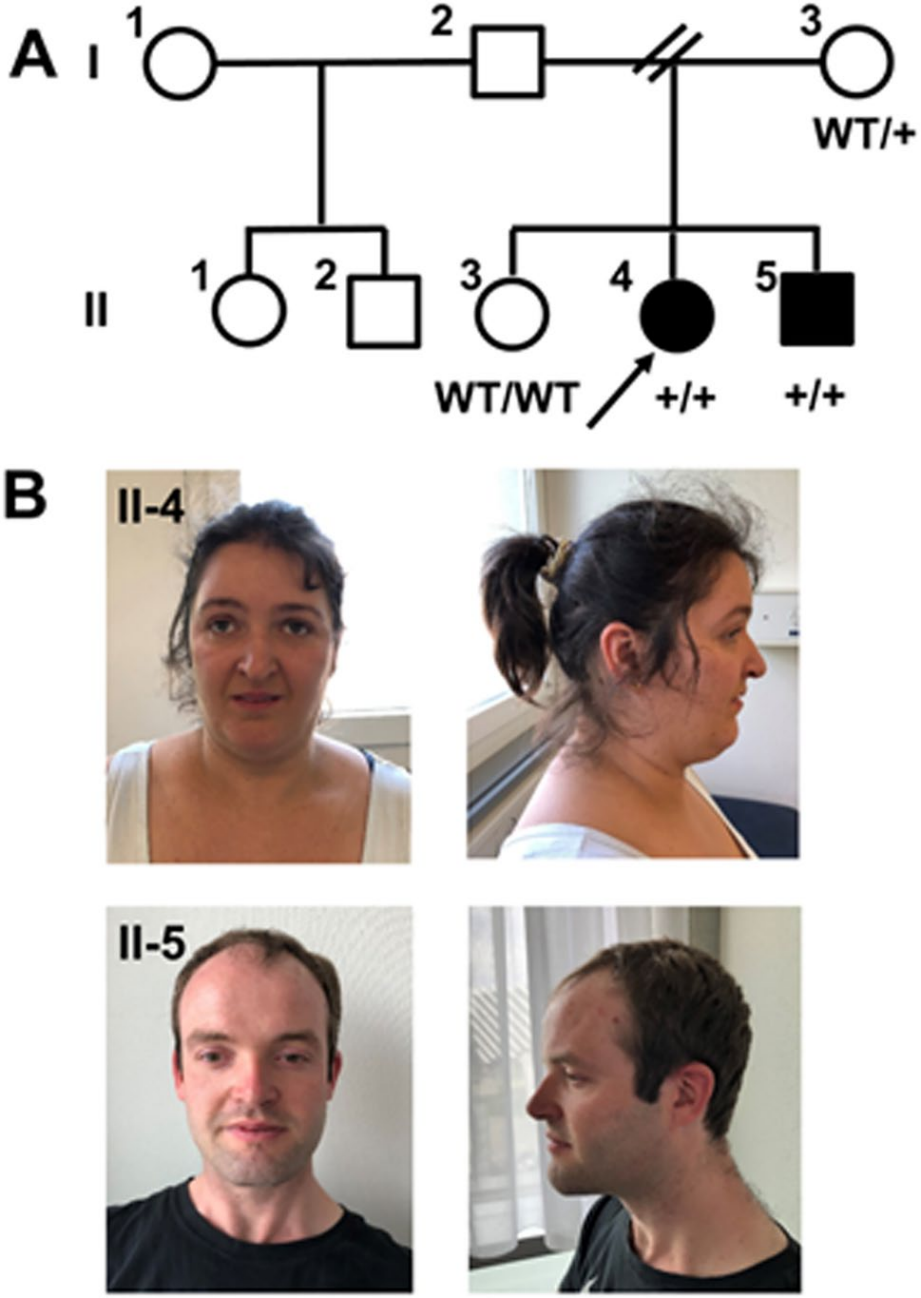
(**A**) Family pedigree. In black, clinically affected individuals. In white, unaffected individuals. WT: wild-type. +: PSP variant. (**B**) Face and profile pictures of the proband II-4 (patient F) and her brother II-5 (patient M).

The proband (II-4, Fig. 2A, patient F) is a 36-year-old female born at 38 weeks to healthy unrelated parents following an uneventful pregnancy. She exhibited good neonatal adaptation (APGAR 9/10/10) and her growth parameters at birth (weight 2890 g (P10-P25), length 47 cm (P10-P25), head circumference 32 cm (P3-P5)) fell within normal range. Her language and motor development were delayed. After achieving independent walking at 19 months, she remained unstable and frequently fell. At the age of three, clinical examination revealed spastic paraparesis with a dystonic component, and she subsequently developed dysarthria. She encountered difficulties at school and received special education. Febrile seizures were reported during infancy, and from the age of 6, she developed epilepsy with generalised tonic–clonic and absence seizures, and myoclonia. At 32 years old, ethosuximide was tapered, and currently her epilepsy is controlled with lamotrigine and topiramate (with compensation for metabolic acidosis induced by topiramate). She still experiences short seizures (absences), especially in the presence of provoking factors. Recent EEGs showed moderate diffuse slowing with bursts of diffuse sharp waves, without any clinical correlation. Brain MRI at age 12 and 19 showed no abnormalities. A neuropsychological assessment at age 29 revealed moderate to severe attention and memory impairment, along with executive dysfunction. In a recent clinical evaluation, physical characteristics included hypoplastic alae nasi, a small mouth and mild retrognathism (Fig. 2B). She exhibited a normal stature (170.5 cm, P75-90), head circumference (54 cm, P10-25), and obesity (96.5 kg, BMI 33.2 kg/m^2^). Her motor phenotype remained relatively stable over time, and despite a spastic and wide-based gait, she is capable of walking independently for short distances. She communicates effectively using sentences and can read simple books. She has very good social skills, and there are no reported behavioural issues. Patient F is currently engaged in sheltered employment and lives independently with her brother, with familial support from the parents.

The 32-years-old younger brother (II-5, Fig. 2A, patient M) of the proband was born at term after an uneventful pregnancy. He adapted well to extrauterine life with birth weight (3230 g), length (50 cm), and head circumference (34.5 cm), lying within the 10–25 percentile range. He began to walk independently at around 14–17 months but experienced delayed language development, uttering his first words at the age of three. Due to learning difficulties, he required special education. Neurological assessments during childhood revealed difficulties in gross and fine motor skills, brisk lower limbs’ reflexes with some degree of hypertonia, and a tendency to toe walk, without frank pyramidal syndrome. At the age of seven, he experienced absence seizures and subsequently developed myoclonus and occasionally, generalised tonic–clonic seizures. Epilepsy is currently successfully managed with a combination of lamotrigine and clobazam; the last seizure (myoclonic) occurred when clobazam was tapered. Recent EEGs showed mild slowing and some burst of sharp waves, without any clinical correlation. Brain MRI at age 17 revealed no abnormalities. A recent evaluation showed mild hypertelorism with no other obvious facial characteristics. He has normal weight and is relatively tall (190 cm, P90). The last neurological assessment revealed dysarthria, saccadic eye pursuit, pyramidal signs in the lower limbs and a spastic gait. Patient M’s motor phenotype, which remained stable over time, is less severe than that of his sister, who experiences significantly more pronounced walking difficulties (also due to obesity). He currently works in a sheltered environment and lives independently with her sister, with support from his family.

The elder sister (II3, Fig. 2A, CTR) exhibits no intellectual disability, motor difficulties or epilepsy; she was used as control reference for the analysis on serum.

### Genetic analysis

Karyotype analysis of the proband and her brother was normal. The array-CGH carried out in the proband did not reveal any loss or gain of genomic material [arr(1–22,X) × 2]. Fragile X syndrome was excluded through genetic testing. Next-generation sequencing in patient F of a large panel of genes related to developmental disorders, hereditary spastic paraplegia, and epilepsy identified the homozygous variant [NM_004577.4: c.398A > G p.(Asn133Ser)] in the *PSPH* gene (OMIM * 172480, the PSP encoding gene). The *PSPH* variant was classified as “variant of unknown significance” due to insufficient evidence to confirm its role in the disease. However, several factors suggest a pathogenic role. Firstly, targeted analysis of the proband’s brother confirmed the presence of the Asn133Ser variant in the homozygous state, while the healthy sister carried two wild-type alleles. The mother was heterozygous for the variant and the father was not available for testing.

The Asn133 residue in PSP is highly conserved across various species, and its substitution with serine is estimated as deleterious by the prediction programs MutScore, REVEL, CADD, VEST4, SIFT, Mutation Taster. Additionally, this amino acid substitution has been observed in heterozygosity at a very low allele frequency (approximately 0.007%) in the population database GnomAD. Interestingly, the homozygous variant Asn133Ser in PSP was recently identified in a 35-year-old patient from a Pakistani consanguineous family with amelogenesis imperfecta due to a biallelic variant in the *SLC24A4* gene^31^. The PSP variant was considered non-causative of the dental disease, and the authors provided no information on other aspects of the patient’s phenotype. The variant has subsequently been reported in the Clinvar database as “variant of unknown significance”, with no details on the patient’s phenotype.

In addition, the next-generation sequencing analysis of patient F identified the heterozygous missense variant [NM_007282.4: c.800T > C p.(Val267Ala)] in the *RNF13* gene (OMIM * 609247), classified as “variant of unknown significance”. The segregation analysis revealed its presence in the affected brother and its absence in the healthy sister and in their mother. A transmission from the healthy father was considered probable but could not be verified. The c.800T > C variant has been reported in the reference population database GnomAD with a very low allele frequency of 0.00012%, but it has never been described in patients to our knowledge. Heterozygous *RNF13* gain-of-function variants have been associated with congenital microcephaly, epileptic encephalopathy, blindness, and failure to thrive. This severe phenotype does not align well with that of the presently described subjects^32^. The contribution of the c.800T > C variant to the patients F and M’s phenotype is considered unlikely but could not be completely excluded.

### Biochemical properties of Asn133Ser PSP

**Figure 3:**
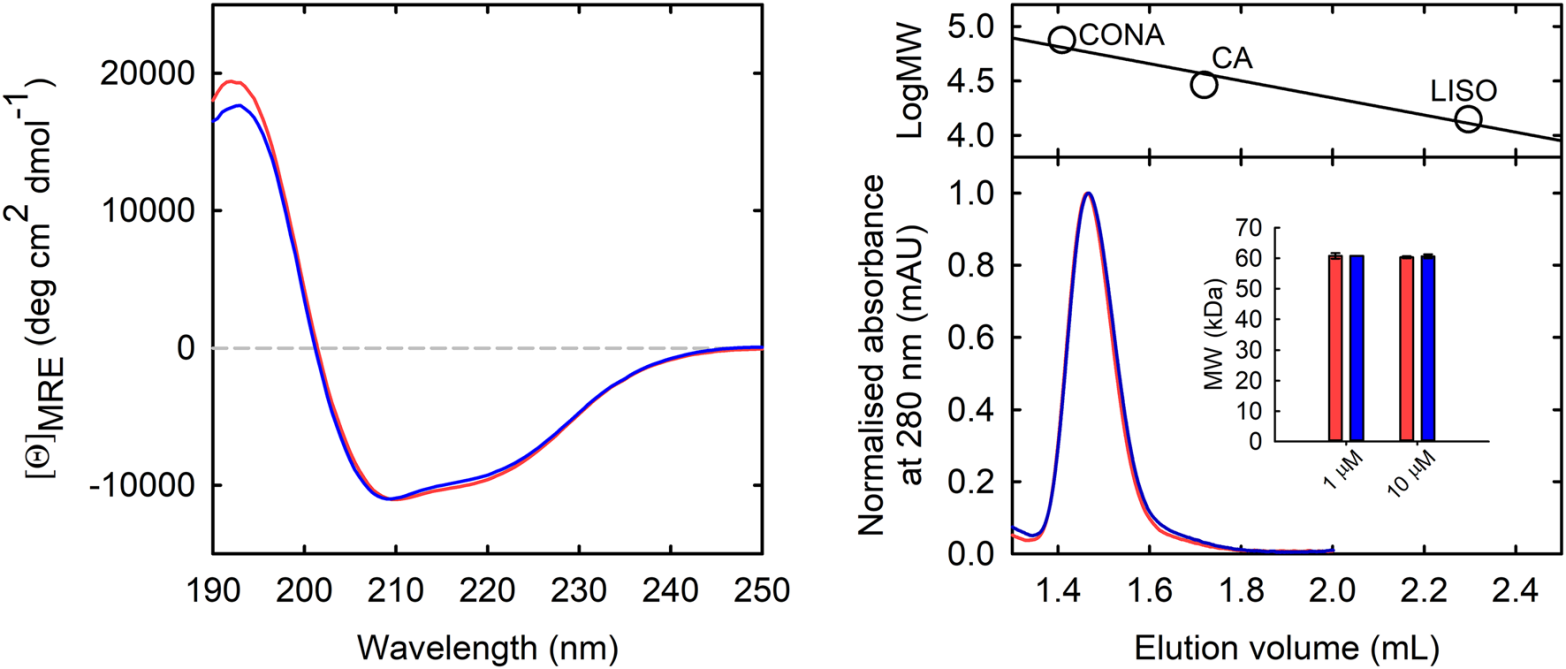
N133S and wt PSP structural properties. (**A**) Circular dichroism spectra of wt (red line) and Asn133Ser PSP (blue line) in 20 mM phosphate buffer, pH 7. (**B**) SEC analysis of either wt (red) and Asn133Ser (blue) PSP at 10 μM concentration in 50 mM Hepes, 100 mM KCl, pH 7, performed on a Superdex 75 Increase column using an ÄKTA Pure System. Inset, the apparent mass of PSP estimated at 1 and 10 μM protein concentration. Upper panel, calibration curve using conalbumin, carbonic anhydrase and lysozyme.

Recombinant Asn133Ser PSP was purified from *E. coli* cells at > 90% purity (not shown). Its specific activity on 3-PS is similar to the one of wt PSP (Table 1). The Asn133Ser substitution does not affect the secondary structure of the protein, as judged from the CD spectra in the far-UV region (Fig. 3A). SEC analysis performed at 10 µM protein concentration showed that both wt and N133S PSP are dimeric, with an apparent molecular mass of 60.3 ± 0.3 kDa and 60.6 ± 0.5 kDa, respectively, consistent with the theoretical mass of 54.3 kDa. Moreover, for both PSP variants the apparent molecular mass estimated at lower protein concentration (i.e. 1 µM) was identical to the one estimated at higher protein concentration, confirming that the substitution does not affect the monomer–dimer equilibrium of PSP (Fig. 3B).

**Table 1:** Kinetic parameters of wild-type and Asn133Ser PSP.

Parameter	wild-type	Asn133Ser
k_cat_ (s^-1^)	38 ± 3	28 ± 1
K_m_ (μM)	42 ± 13	31 ± 5
k_cat_/K_m_ (M^−1^ s^−1^)	0.9 × 10^6^	0.9 × 10^6^
Specific activity (U/mg protein)	82	62
IC_50_ (L-Ser) (μM)	290 ± 20	250 ± 25

The assays were carried out at 37 °C in 50 mM HEPES, 100 mM KCl, 3 mM MgCl_2_, pH 7. The values are reported ± standard error.

**Figure 4:**
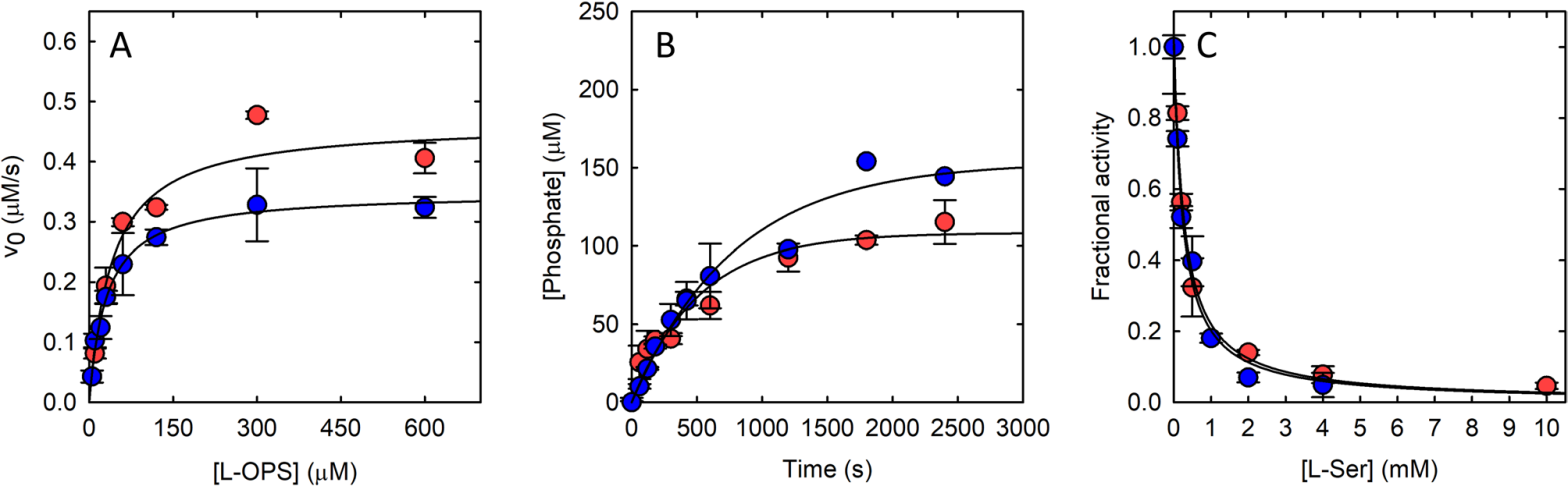
Activity of Asn133Ser (blue symbols) and wild-type (red symbols) PSP. (**A**) Dependence of initial velocity on 3-PS (L-OPS) concentration. The solid line represents fitting to Eq. 1 with k_cat_ and K_m_ values reported in Table 1. (**B**) Time course of phosphate production by the in vitro reconstructed phosphorylated pathway. (**C**) Dependence of the relative activity of PSP on L-Ser concentration. The solid lines represent fitting to Eq. 2 with IC_50_ values reported in Table 1.

The kinetic parameters of Asn133Ser PSP do not significantly differ from those of the wt counterpart, with only a slight reduction of k_cat_ that is compensated by a decrease in K_m_ to give an identical catalytic efficiency (Fig. 4A, Table 1). Notably, the physiological concentration of 3-PS, about 5 μM^2^, is significantly lower than the K_m_ of PSP for its substrate, thus the enzyme works under k_cat_/K_m_ regime and the substitution is not expected to affect the overall rate of L-Ser production. This is confirmed by the observation that when the enzyme is evaluated within the reconstructed metabolic pathway^2^, no effect on the production of phosphate (and thus of L-Ser) is observed (Fig. 4B).

The IC_50_ for the inhibition by the final product L-Ser is also unaffected by the substitution (Fig. 4C).

Overall, the PSP variant seems able to sustain L-Ser production similarly to the wt PSP. The reduction of L-Ser measured in the blood and fibroblasts of the patients carrying the mutation (see below) cannot be ascribed to an effect of the Asn133Ser substitution on the catalytic activity of the enzyme or the functionality of the serinosome metabolon.

### Stability of Asn133Ser PSP

**Figure 5:**
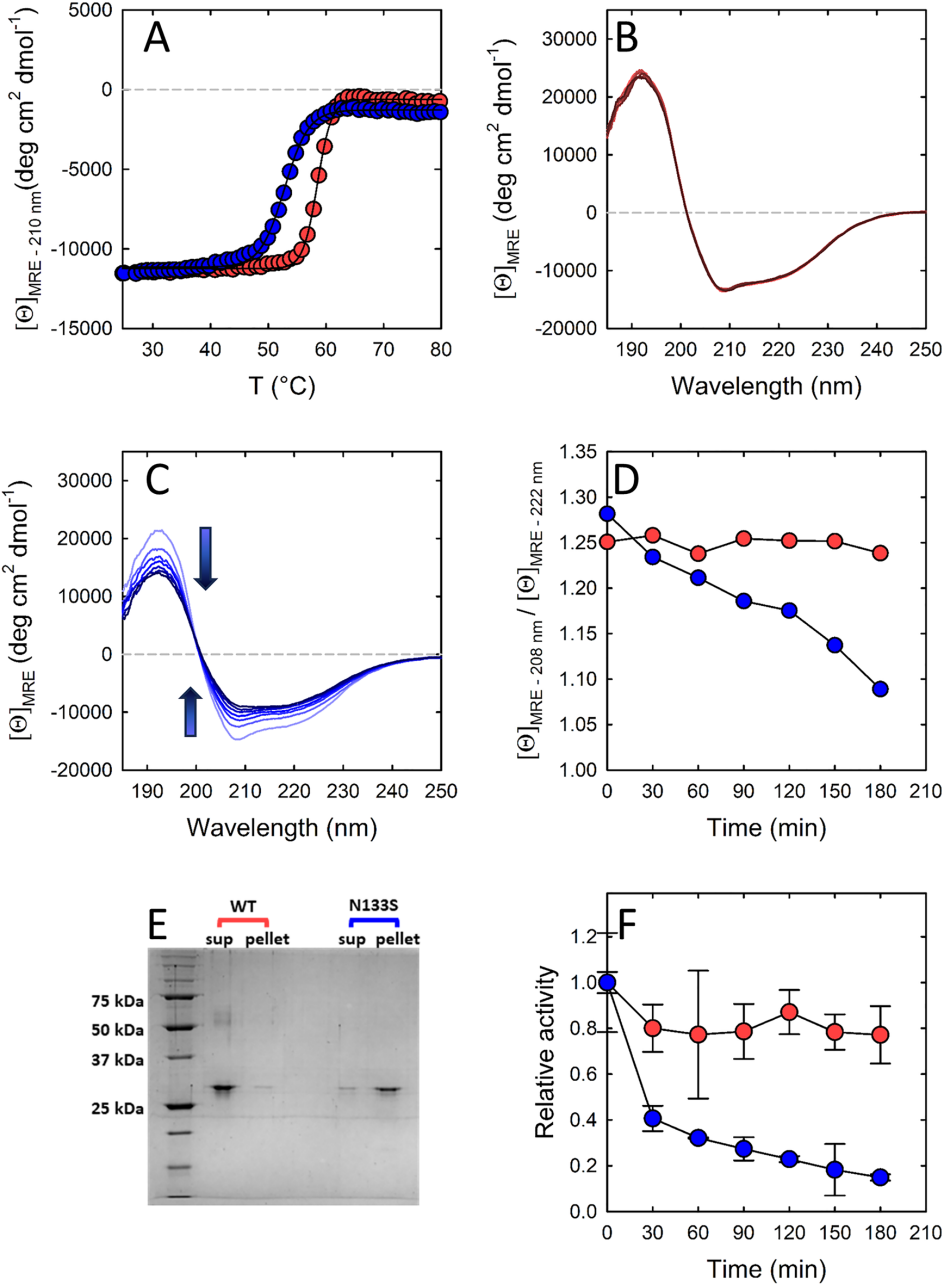
Stability of Asn133Ser (blue symbols) and wild-type (red symbols) PSP. (**A**) Thermal stability in the presence of 0.125 mM MgCl_2_. Lines represent the fitting to Eq. (3): T_m_ = 58.5 ± 0.1 °C and k = 1.23 ± 0.04 for wt, and T_m_ = 52.8 ± 0.1 and k = 2.09 ± 0.07 for N133S PSP. (**B**, **C**) Stability of the secondary structure of either wt (**B**) or Asn133Ser (**C**) PSP on incubation at 37 °C in the presence of 0.125 mM MgCl_2_. (**D**) The ratio between the ellipticity at 210 nm and 222 nm from panels B and C was plotted as a function of time. (**E**) SDS-PAGE of the soluble (sup) and insoluble (pellet) fractions of wt and Asn133Ser PSP following incubation at 37 °C for 3 h in the presence of 0.125 mM MgCl_2_. (**F**) Time-course of PSP activity during incubation at 37 °C in the presence of 0.125 mM MgCl_2_ and either wt or Asn133Ser PSP. The activity value before incubation is reported as 1.

The thermal stability of wt and Asn133Ser PSP was assessed by measuring the disappearance of the secondary structure of the protein upon thermal unfolding using CD spectroscopy. The assay was carried out in the presence of 0.125 mM MgCl_2_ (lower limit of physiological concentration in human astrocytes) considering that Mg^++^ is a known ligand and activator of PSP (Fig. 5A)^810,33^. The melting temperature (T_m_, calculated using Eq. 3) of Asn133Ser PSP is 52.8 ± 0.1 °C, significantly lower than the T_m_ of wt PSP (58.5 ± 0.1 °C), thus suggesting a possible destabilising role of the substitution on PSP structure. Furthermore, the slope of the thermal transition for the Asn133Ser variant is significantly lower than the slope for the wt enzyme, further confirming a destabilising effect of the substitution that is associated with a less cooperative unfolding process^34^.

To further investigate the effect of the Asn133Ser substitution on the enzyme stability, the enzyme was incubated at 37 °C and CD spectra were collected every 30 min over a 3 h period. At the end of the experiment the solution containing the enzyme was centrifuged and the protein fractions in the supernatant and in the pellet were analysed by SDS-PAGE (Fig. 5B–E). The incubation was carried out in potassium phosphate buffer pH 7.0, 0.125 mM MgCl_2_ to better mimic a physiological condition. The secondary structure of wt PSP does not change during incubation (Fig. 5B), while the CD signal of the Asn133Ser PSP decreases with time (Fig. 5C). Furthermore, also the ratio between the ellipticity at 208 nm and 222 nm changes (Fig. 5D), an indication of alteration in the content of the secondary structure of the protein. The decrease in signal intensity is in part due to protein aggregation and precipitation, as demonstrated by the SDS-PAGE results: after centrifugation of the reaction mixture incubated for 3 h at 37 °C, wt PSP is largely present in the soluble fraction, while the Asn133Ser variant is mostly in the pellet (Fig. 5E).

The same trend is apparent when the residual enzymatic activity is recorded during incubation at 37 °C in the presence of 0.125 mM MgCl_2_ (Fig. 5F). While the activity of wt PSP is quite stable, the activity of Asn133Ser PSP decreases with time: after 3 h of incubation, the wt enzyme retains about 80% of the initial activity compared to a 20% for the Asn133Ser variant.

### Serine and glycine levels in serum and fibroblasts of affected patients

**Figure 6:**
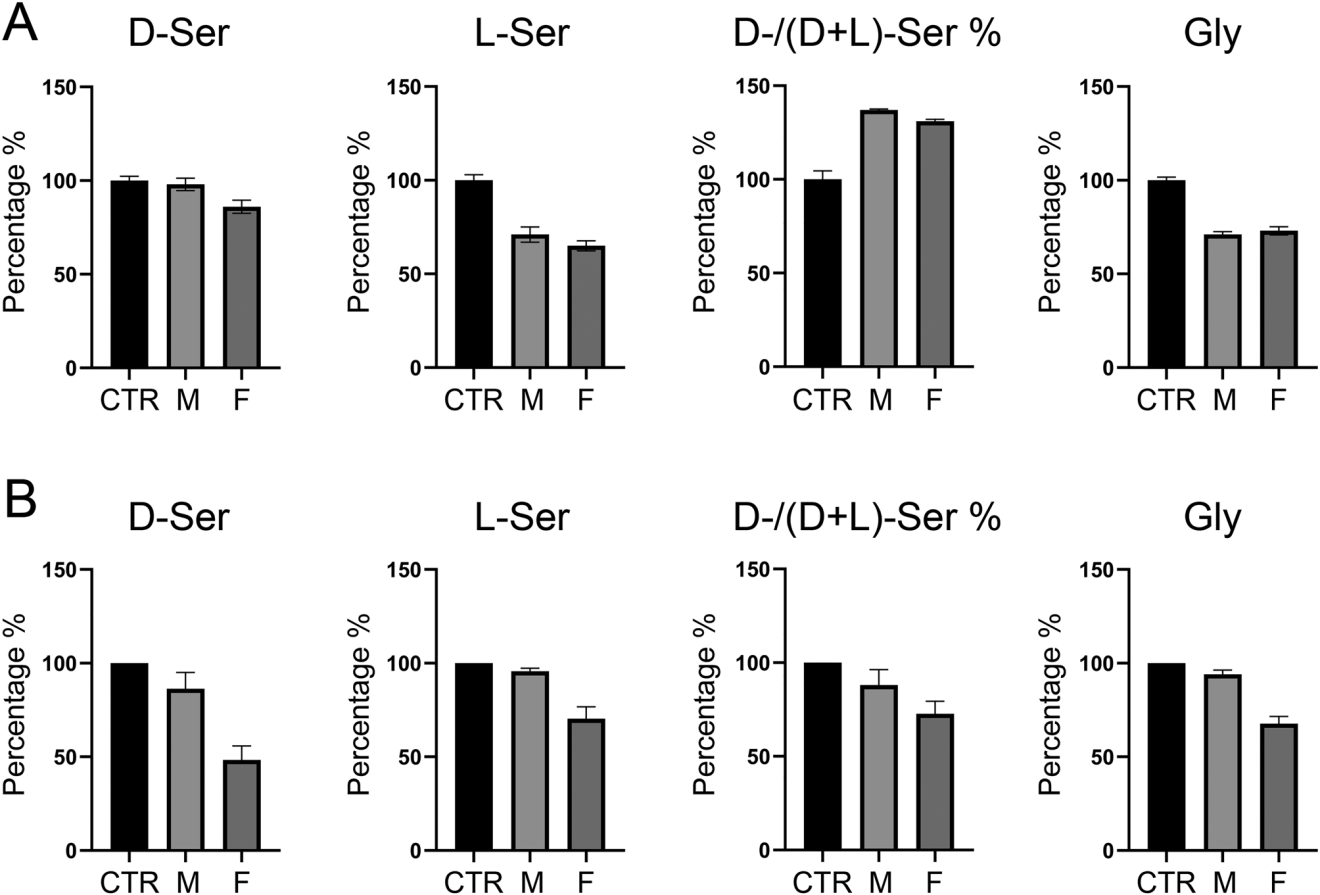
Levels of D-Ser, L-Ser and Gly in serum and cultured fibroblasts samples. (**A**) D-Ser, L-Ser, D-/(D + L)- Ser ratio and Gly levels in serum samples from patients and healthy control expressed as percentage considering CTR sample as 100%. Graphs report the mean values ± SEM of at least 3 HPLC runs for each sample. (**B**) D-Ser, L-Ser, D-/(D + L)-Ser ratio and Gly levels in cultured fibroblasts samples from patients and healthy control expressed as percentage considering CTR sample as 100%. Graphs report the mean values ± SEM of 3 cells samples at different times (p6, p9, p12) each of one analysed in at least 3 technical replicates. Concentration values are reported in Suppl. Table 1.

In order to verify whether this PSP variant affects serine and glycine levels, serum and cultured fibroblast samples were analysed by HPLC analysis^27^: the concentration values are shown in Suppl. Table 1 while the relative amount, expressed as percentages considering the values corresponding to the CTR sample (the healthy sister for the blood sample) as 100%, is depicted in Fig. 6. A decreased L-Ser level in patients’ samples was observed in both serum and cellular samples (− 29.3% for M and − 35.2% for F; − 4.2% for M and − 29.9% for F, respectively). This reduction is associated with a decrease in D-Ser levels that is more evident in fibroblasts (− 13.7% and − 51.9% for M and F, respectively) than in serum (− 1.6% and − 13.9% for M and F, respectively). The serine level in serum is known to be strongly dependent on nutrition, while endogenous synthesis plays a relevant effect in the brain because of the low permeability of this amino acid through the blood brain barrier^35^. Interestingly, the highest decrease in D-Ser levels is evident for the F patient even in serum samples. These data lead to a significant decrease for the D/(D + L)-Ser ratio in fibroblasts samples and an increase in serum (Fig. 6). A significant decrease in Gly levels was evident for both serum (− 29.0% for M and − 26.6% for F) and cellular (− 6.0% for M and − 32.5% for F) samples compared to CTR. The concentration values for the selected amino acids recently determined in the blood of 11 healthy individuals by the same analytical procedure^36^ show a good agreement with the values determined for the healthy control, see Suppl. Table 1.

### The serinosome in patients’ fibroblasts: enzymes level and colocalization

**Figure 7:**
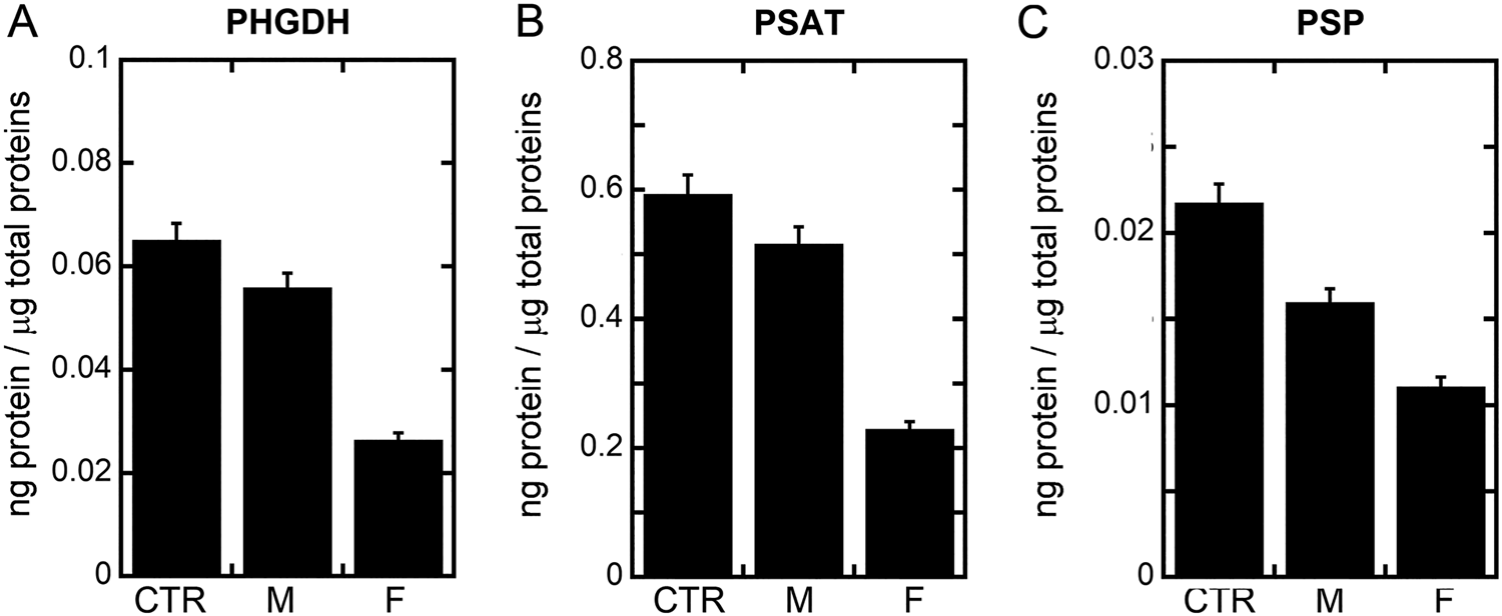
Comparison of PHGDH (**A**), PSAT (**B**) and PSP (**C**) levels in fibroblast samples from affected patients and a healthy control. Bars represent the mean ± SEM of values obtained from 3 sets of samples.

The expression levels of PHGDH, PSAT, and PSP in fibroblasts from affected patients and a healthy control (unrelated) were determined by Western blot analysis (Suppl. Fig. 1). As reported by the densitometric analysis, in all samples PSAT showed the highest expression levels, ~ 10–20-fold higher than the ones for PHGDH and PSP (Fig. 7). Both patients showed reduced expression levels of the three proteins of the PP compared to control (Fig. 7). The Asn133Ser substitution in PSP affects the expression level of PSP as well as of the other two proteins (PHGDH and PSAT) of the metabolism of L-Ser, in particular in the case of the F patient, also hampering serine production (see above).

The cellular stability of PHGDH, PSAT and PSP was investigated by measuring the cellular level of each enzyme following the treatment for up to 24 h of fibroblasts with cycloheximide (CHX), an inhibitor of protein synthesis that prevents translational elongation. In control fibroblasts, the three enzymes of the PP are long-life proteins. Patients show a slight alteration in the half-life of PHGDH and PSAT compared to the control, while this alteration is most apparent for PSP (Table 2, Suppl. Fig. 2).

**Table 2:** Half-life values of PHGDH, PSAT and PSP in fibroblasts from affected patients (M and F) and a healthy control (CTR).

t_1/2_ (min)			
Sample	PHGDH	PSAT	PSP
CTR	1125 ± 135	2300 ± 230	≥ 3000
M	1300 ± 145	1625 ± 310	900 ± 90
F	1200 ± 145	1600 ± 200	1200 ± 150

The following results are based on data obtained from three separate experiments. Values are reported as mean ± standard error.

In conclusion, the observed reduction in expression levels of the PP enzymes in fibroblasts expressing the Asn133Ser PSP variant does not seem to be due to a decrease in protein half-life in the case of PHGDH and PSAT, while the lower stability of variant PSP affects its cellular level.

**Figure 8:**
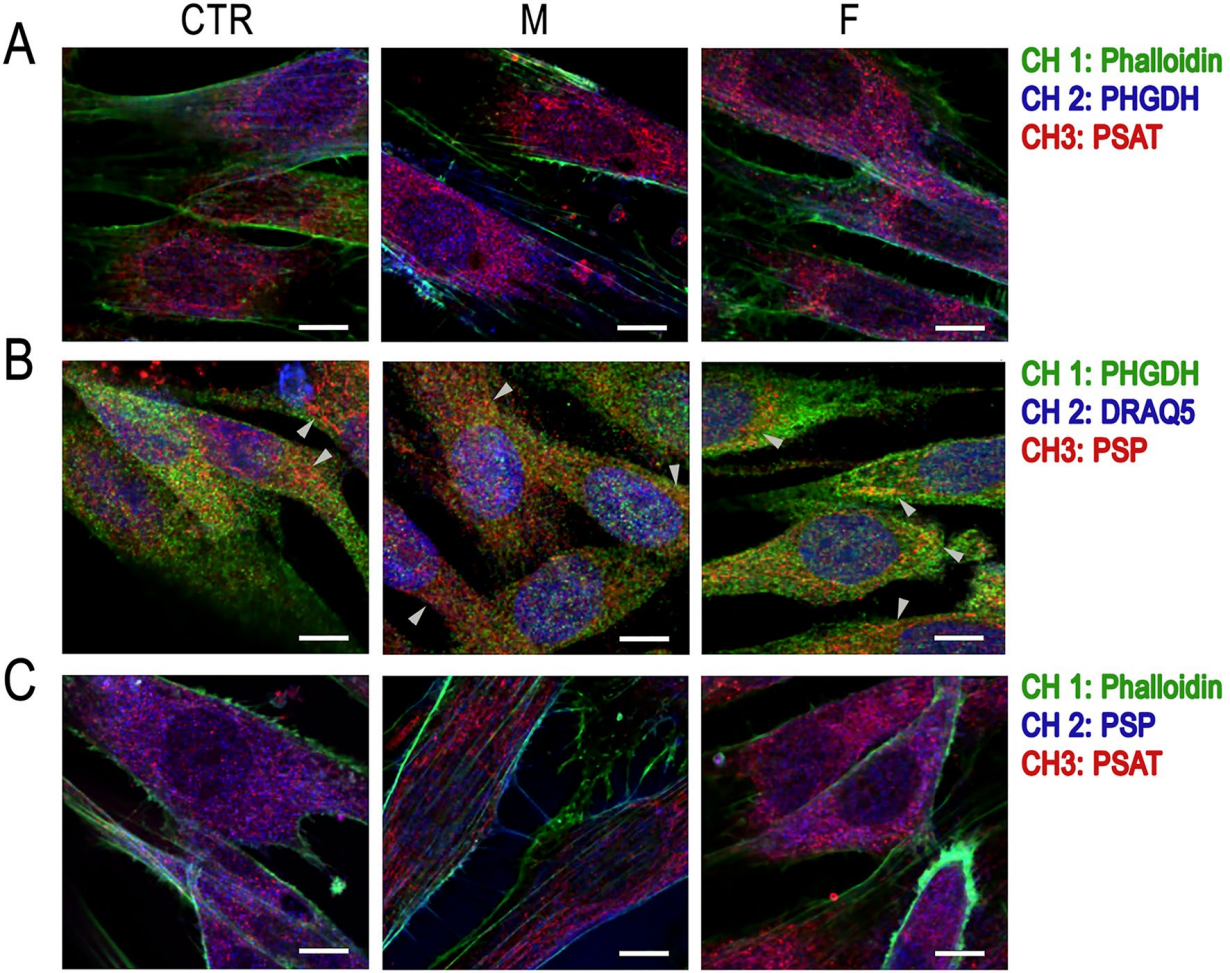
PHGDH, PSAT and PSP cellular localization in CTR and M and F patient-derived fibroblasts, as detected by immunofluorescence and confocal analysis. Cells were double stained using rabbit anti-PHGDH + mouse anti-PSAT (**A**), mouse anti-PHGDH + rabbit anti-PSP (**B**), and mouse anti-PSAT + rabbit anti-PSP (**C**) antibody mixtures and counterstained with phalloidin (labelling the cytoskeleton) and DRAQ5 (a fluorescent and high affinity nuclear dye). Scale bar = 7.5 µm. Arrowheads indicate protein aggregates.

The effect of Asn133Ser PSP pathological substitution on the cellular distribution of the PP enzymes in the patient-derived fibroblasts and on their ability to organise in a metabolic assembly, the serinosome^2^, was investigated by double staining immunofluorescence and confocal analyses. In general, PHGDH, PSAT and PSP showed a predominant cytosolic localization with an apparent perinuclear accumulation and low levels of the corresponding signals observed in the nucleus (Fig. 8). Colocalization analyses showed that the signals corresponding to the PP enzymes partially overlapped within the fibroblasts of the control (CTR) and the M and F patients, as indicated by the related coefficients that measure the co-occurrence of signals (M1 and M2, Suppl. Table 2). Most importantly, a partial but significant colocalization of couples of signals corresponding to PHGDH:PSAT, PHGDH:PSP and PSAT:PSP was evident for CTR based on the estimated Pearson’s coefficient (*r*), in the 0.35–0.50 range. Notably, lower *r* values were determined for the patient M (0.37 ± 0.1, 0.26 ± 0.03 and 0.18 ± 0.03 for PHGDH:PSAT, PHGDH:PSP and PSP:PSAT, respectively), while slight changes were apparent between patient F and CTR (with *r* values in the range 0.4–0.45, Suppl. Table 2).

In line with the recently reported observations in NPC-derived differentiated astrocytes, suggesting that PHGDH is less frequently and/or less stably associated within the protein clusters^2^, a higher and largely distributed colocalization of PSAT and PSP signals is evident in control cells. On the other hand, lower *r* values and colocalization of signals were apparent for the M patient (Fig. 8B,C and Suppl. Table 2) and for the F patient relatively to the PSP:PSAT colocalization only. In this latter patient the cellular content of all PP proteins is approximately halved (Fig. 7) thus the molar ratio is conserved while actual levels are decreased; on the other hand, in the M patient the PSP level only is substantially decreased (1.4-fold compared to CTR), this possibly accounting for the detected stronger decrease in colocalization.

In few control cells the distribution of PSP signal likely indicates the formation of cytosolic aggregates (arrowheads, Fig. 8B left panel), a trend that is markedly most evident in patient-derived fibroblasts, where the large majority of cells exhibit these PSP clusters (Fig. 8B,D central and right panels). In the latter cells, the clustering of the PSP variant apparently affects PSAT signal pattern: while PSAT is mainly diffused in the cytosol of control cells, it shows the formation of large perinuclear aggregates in M and F patients-derived fibroblasts, partly overlapping to PSP signal (Fig. 8C).

Altogether, these data suggest that the expression of the Asn133Ser PSP variant, beside decreasing the level of expression of the PP enzymes, promotes the aggregation of the pathological variant as well as of PSAT, likely yielding to the formation of a dysfunctional serinosome.

## Discussion

Based on the results obtained on the recombinant enzyme, the Asn133Ser substitution in human PSP does not significantly affects the activity of the isolated enzyme or the overall activity of the in vitro reconstructed PP, as well as the protein secondary structure composition and the dimeric oligomerization state. However, the Asn133Ser variant is less stable than wild-type PSP (a 6 °C decrease in T_m_ is observed), and this feature is also apparent at the cellular level: its half-life is fivefold lower compared to wild-type PSP, see Table 2. Studies on patients’ fibroblasts also highlight (Fig. 7 and 8): (i) a strong decrease in the level of the three enzymes of the PP, especially for the F patient; (ii) a partial nuclear and perinuclear localization of variant PSP; (iii) a partial, and higher than wt PSP, signal clustering with PHGDH and PSAT (less evident for the M patient); (iv) a stronger perinuclear aggregates formation. The decrease in the cellular concentration of the PP enzymes and the formation of aggregates containing variant PSP and PSAT contribute to the formation of a dysfunctional serinosome and thus might lead to a substantial reduction of L-Ser production in the brain, thus explaining the lower concentration of this amino acid found in the patient’s serum and fibroblasts. Notably, the decrease in Gly levels parallels the ones observed for L-Ser (Fig. 6), clearly demonstrating the strict correlation between these two amino acids via the SHMT pathway. Indeed, the D-Ser levels in the F patient fibroblasts are > twofold decreased compared to CTR; the low cellular amount of this NMDA receptor co-agonist could be relevant to rationalise the more drastic clinical conditions of such a patient. Furthermore, HPLC enantiomeric measurements highlight that the effect of the pathological mutation in terms of amino acid level’s alteration is better appreciated on fibroblasts than on serum: notably, the measured amino acids values in the control show excellent agreement with literature data from healthy individuals^36^.

This study suffers from two main limitations: (a) an influence on the clinical phenotype by another genetic variant, which may have gone undetected by the presently analysed gene panel, is not entirely ruled out. In particular, a phenotypic impact of the Val267Ala variant in the *RNF13* gene could not be definitively excluded, though it is considered improbable based on the reasons explained above; (b) the reference control, even if well matched for the blood analyses (i.e. the healthy sister), is represented by a single sample: physiological alterations in PSP cellular levels as well as alterations in L-Ser levels due to diet cannot be excluded.

## Conclusions

The biochemical and cellular characterization of patients harbouring the Asn133Ser PSP substitution suggests the use of L-Ser supplementation as add-on to medications with the aim to recover from the observed L-Ser deficit (as well as D-Ser and Gly deficits) pointing, at the same time, to propose novel targeted treatments to cope with protein aggregation and to protect brain D-Ser from D-amino acid oxidase-induced degradation^37^.

### Supplementary Information

Supplementary Information.

## Data Availability

Data supporting this study are available from the authors (Loredano Pollegioni, Barbara Campanini and Jean-Marc Good) on request.
