# Supplementary material for: L-serine deficiency: on the properties of the Asn133Ser variant of human phosphoserine phosphatase
Source: Sci Rep. 2024 May 30;14:12463. doi: 10.1038/s41598-024-63164-y (PMC11139964; doi:10.1038/s41598-024-63164-y)
Supplement: Supplementary file 1 — Supplementary Information. [file 41598_2024_63164_MOESM1_ESM.pdf]

Supplemental Data

L-SERINE DEFICIENCY: ON THE PROPERTIES OF THE Asn133Ser VARIANT OF HUMAN PHOSPHOSERINE PHOSPHATASE

Loredano Pollegioni[1*§], Barbara Campanini[2*], Jean-Marc Good[3*], Zoraide Motta[1], Giulia Murtas[1], Valeria Buoli Comani[2], Despina-Christina Pavlidou[3], Noëlle Mercier[4], Laureane Mittaz-Crettol[3], Silvia Sacchi[1], Francesco Marchesani[5]

[1]Department of Biotechnology and Molecular Sciences, University of Insubria, via J.H. Dunant 3, 21100 Varese (Italy)

[2]Department of Food and Drug, University of Parma, 43124, Parma, Italy.

[3]Division of Genetic Medicine, University of Lausanne and University Hospital of Lausanne, Lausanne, Switzerland

[4]Department of Epileptology, Institution of Lavigny, Lavigny, Switzerland.

[5]Department of Medicine and Surgery, University of Parma, 43121, Parma, Italy

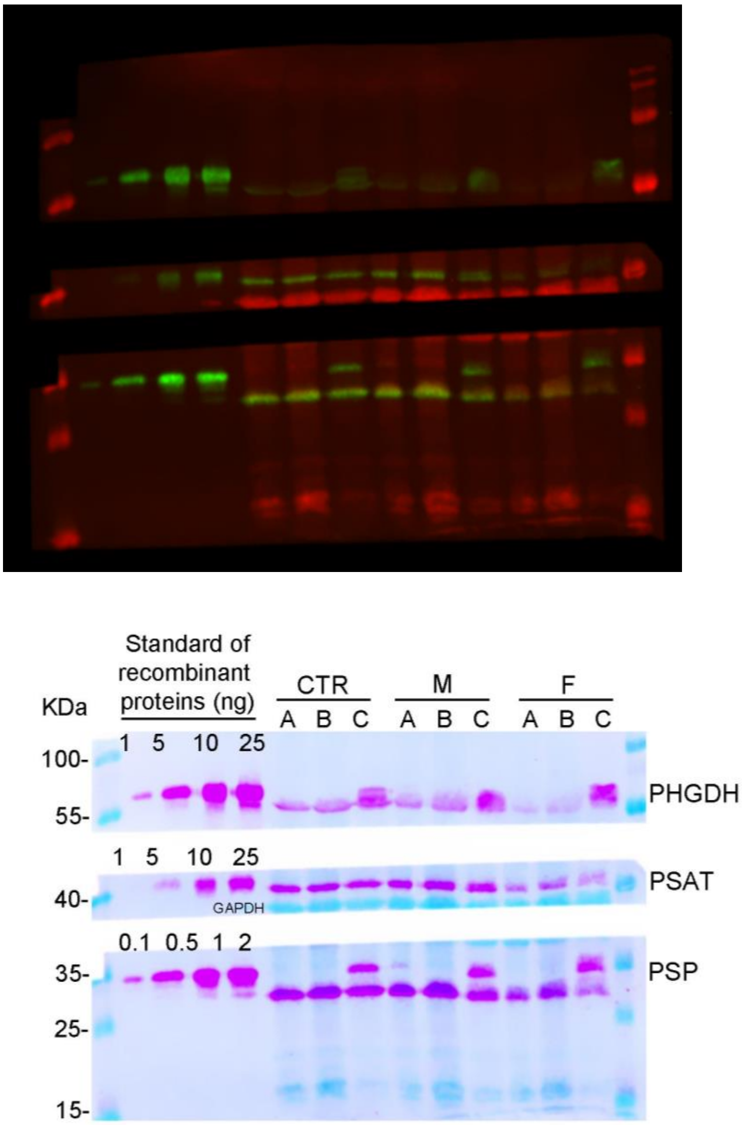

Supplementary Figure 1. Western blot analysis of the levels of the PP enzymes in fibroblasts of patients M and F and a healthy control (CTR). The membrane was cut into three pieces: top: 150–50 kDa range, containing PHGDH, 56.7 kDa; center: 36–50 kDa range, containing PSAT and glyceraldehyde-3-phosphate dehydrogenase (GAPDH, used as loading control), 40.5 and 37 kDa, respectively; bottom: 23–36 kDa range, containing PSP (25 kDa. A: 20 µg total proteins: B: 40 µg total proteins: C: 20 µg total proteins added of recombinant standard proteins (5 ng for PHGDH or PSAT, 0.5 ng for PSP).

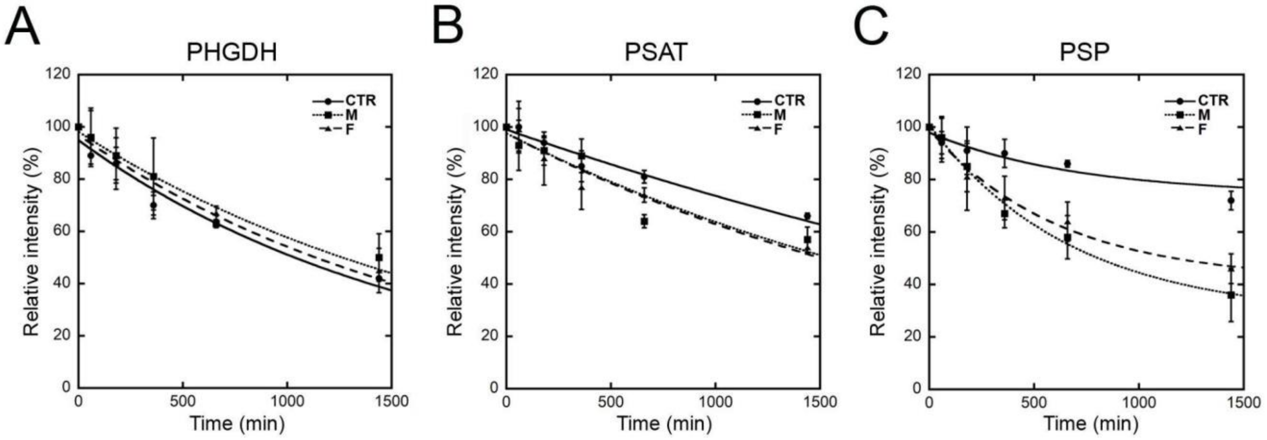

Supplementary Figure 2. Half-life analysis of PHGDH (A), PSAT (B) and PSP (C) in human fibroblasts from a healthy control (CTR, black) and affected patients (M and F dotted and dashed line, respectively). The fitting of experimental data was carried out using a single exponential decay.

Supplementary Table 1. Concentration serum and fibroblasts of amino acids measured by HPLC analysis.

Top: values represent the mean of at least 3 runs of each sample ± standard deviation of serum samples. Concentration values are expressed in µM. Bottom: values represent the mean of concentrations of samples from p6, p9 and p12 expressed in nmol of amino acids normalized on total protein content of the sample ± standard deviation.

Fibroblasts	**D-Ser (nmol/mg protein)**	**L-Ser (nmol/mg protein)**	**D-/(D+L)-Ser (%)**	**Gly (nmol/mg protein)**
**CTR**	0.059±0.022	16.8±8.4	0.387±0.055	76.4±46.6
**M**	0.052±0.027	16.2±8.5	0.335±0.010	71.8±43.9
**F**	0.028±0.010	12.0±7.3	0.281±0.025	50.3±30.6

Serum	**D-Ser (µM)**	**L-Ser (µM)**	**D-/(D+L)-Ser (%)**	**Gly (µM)**
**CTR**[a]	1.01±0.04 (1.24±0.35)	109.0±5.3 (147±33)	0.92±0.08 (0.84±0.15)	198.4±6.8 (161±29)
**M**	0.99±0.07	77.1±5.4	1.26±0.01	140.8±4.3
**F**	0.87±0.06	70.7±4.2	1.20±0.02	145.6±7.8

[a] in parenthesis is reported the value determined in blood samples of 11 healthy patients in [36].

Supplementary Table 2. Colocalization analysis.

Parameter related to signal co-occurrence, i.e the Manders' overlap (M1 and M2) coefficients, as well as to signals correlation/colocalization, i.e. Pearson's correlation coefficient (*r*), were determined by Fuji (ImageJ) open access software. The reported values refer to the analysis of signals corresponding to couples of PP proteins and the immunostainings in the corresponding panels of Figure 8 (PHGDH and PSAT; PHGDH and PS; PSP and PSAT, panels A, B and C, respectively).

Couples	CTR			M			F		
	M1	M2	r	M1	M2	r	M1	M2	r
**PHGDH PSAT**	0.57±0.2	0.58±0.1	0.39±0.2	0.57±0.1	0.66±0.1	0.37±0.12	0.54±0.1	0.69±0.1	0.46±0.06
**PHGDH PSP**	0.65±0.03	0.55±0.03	0.36±0.1	0.62±0.1	0.30±0.1	0.26±0.03	0.68±0.1	0.64±0.1	0.44±0.06
**PSP PSAT**	0.59±0.1	0.75±0.1	0.50±0.2	0.34±0.03	0.51±0.1	0.18±0.03	0.53±0.1	0.68±0.1	0.41±0.03
